# Evaluating the most appropriate pooling ratio for EDTA blood samples to detect Bluetongue virus using real-time RT-PCR

**DOI:** 10.1016/j.vetmic.2018.03.001

**Published:** 2018-04

**Authors:** John Flannery, Paulina Rajko-Nenow, Hayley Hicks, Holly Hill, Simon Gubbins, Carrie Batten

**Affiliations:** The Pirbright Institute, Pirbright, Woking, Surrey, United Kingdom

**Keywords:** Bluetongue virus, Real-time RT-PCR, Surveillance, European serotypes, VetMAX

## Abstract

The control of Bluetongue virus (BTV) presents a significant challenge to European Union (EU) member states as trade restrictions are placed on animals imported from BTV-affected countries. BTV surveillance programs are costly to maintain, thus, pooling of EDTA blood samples is used to reduce costs and increase throughput. We investigated different pooling ratios (1:2, 1:5, 1:10 and 1:20) for EDTA blood samples to detect a single BTV positive animal. A published real-time RT-PCR assay (Hofmann et al., 2008) and a commercial assay (ThermoFisher VetMax™ BTV NS3 kit) were used to analyse BTV RNA extracted from pooled EDTA blood samples. The detection rate was low for the onset of infection sample (0–2 days post infection (dpi); C_T_ 36) irrespective of the pooling ratio. Both assays could reliably detect a single BTV-positive animal at early viraemia (3–6 dpi; C_T_ 33) when pooled, however, detection rate diminished with increasing pooling ratio. A statistical model indicated that pooling samples up to 1:20, is suitable to detect a single BTV positive animal at peak viraemia (7–12 dpi) or late infection (13–30 dpi) with a probability of detection of >80% and >94% using the Hofmann et al. (2008) and VetMAX assays, respectively. Using the assays highlighted in our study, pooling at ratios of 1:20 would be technically suitable in BTV-endemic countries for surveillance purposes. As peak viraemia occurs between 7–12 days post infection, a 1:10 pooling ratio is appropriate for post-import testing when animals are sampled within a similar time frame post-import.

## Highlights

• BTV is detectable up to 30 days post infection in ruminants.
• Pooling samples at ratios >1:10 is suitable for surveillance in BTV-endemic areas.
• Peak BTV viraemia occurs between 7–12 d.p.i.
• Pooling at ratios >1:10 is suitable when animals are sampled 7–10 days post import.

## 1 Background

Bluetongue (BT) is an infectious haemorrhagic disease of ruminants, caused by bluetongue virus (BTV) of the genus Orbivirus within the family Reoviridae (Maclachlan et al., 2009). BTV is a serologically and genetically diverse virus that is transmitted between ruminant hosts via Culicoides biting midges (Carpenter et al., 2013). BT is a notifiable disease and trade restrictions are placed on countries which have active BTV infection within EU member states (Anonymous, 2000). Since 2007, a variety of BTV serotypes (BTV-1, BTV-2, BTV-4 BTV-8, BTV-9, and BTV-16 have circulated within the EU and have incurred animal movement restrictions (Belbis et al., 2017; Niedbalski, 2015). In addition to these “typical” BTV serotypes, BTV-25, BTV-26 and BTV-27 represent a novel clade of serotypes which circulate predominately in goats but which unlike other BTV serotypes, can be transmitted directly (Batten et al., 2014; Bréard et al., 2018). Although vaccines are available for BTV, only a limited number of inactivated BTV vaccines (BTV-1, BTV-2, BTV-4 and BTV-8) are approved for use within the EU (More et al., 2017). Therefore, control measures for BTV largely rely on animal movement restrictions and appropriate surveillance and testing programmes.

Real-time RT-PCR is the main diagnostic test for the detection of BTV in ruminants and allows competent authorities to control animal trade within the EU. The significant cost of BTV surveillance programmes represents a financial burden for member states and their respective diagnostic laboratories (Pinior et al., 2015). To reduce some of the costs associated with surveillance programmes, pooling of EDTA blood samples is practiced in a number of laboratories (Vandenbussche et al., 2008a). However, the most appropriate pooling ratio to allow for the detection of a single BTV-positive animal within a pool has not been fully determined. A previous report (Vandenbussche et al., 2008a) found that a large percentage of samples containing high C_T_ values (C_T_ > 35) would not be detected using a pooling ratio (defined as 1/ n where n is the number of samples constituting the pool) of 1:10 if animals were at an early stage of viraemia. Another study found that pooling of samples taken at peak viraemia would allow for detection of a BTV–positive animal but that pooling of samples taken at onset of infection would reduce the detection ability of the assay (Batten et al., 2009). The real-time RT-PCR assays investigated in both studies (Shaw et al., 2007; Toussaint et al., 2007) were designed to detect BTV serotypes 1–24, however, since that time a number of new serotypes have been identified (Hofmann et al., 2008; Schulz et al., 2016) which may present a detection challenge for those assays.

Recently, a number of real-time RT-PCR assays have been recognised as being broadly-reactive whilst achieving the greatest sensitivity and have thus been adopted by a large proportion of BT-testing laboratories. Within its remit as European Union Reference Laboratory for BT, The Pirbright Institute organises an annual inter-laboratory proficiency test (PT) to assess the diagnostic assays in use in EU member state national reference laboratories (NRLs) and representative laboratories of third countries. During the 2016 PT, the Hofmann et al. (2008) assay and the commercial assay ThermoFisher VetMAX™ BTV NS3 kit (hereafter: VetMAX assay) were identified as being the most-widely used whilst yielding the greatest analytical sensitivity.

This study describes an investigation into the most appropriate pooling ratio for EDTA blood samples to allow for the detection of a single BTV infected animal at four different stages of BTV infection. The C_T_ values for onset of infection C_T_ 36 (0–2 dpi), early viraemia C_T_ 33 (3 to 6 dpi), peak viraemia C_T_ 27 (7 to 12 dpi) and late infection C_T_ 30 (13–30 dpi) were ascribed based on a review of BTV animal infection studies. EDTA blood samples containing BTV levels representative of different stages of BTV infection were pooled at 1:2, 1:5, 1:10 and 1:20 ratios and tested using the Hofmann et al. (2008) and VetMAX assays. The results from our study would support a risk-based rationale for using various pooling ratios in different BTV epidemiological situations.

## 2 Methods

### 2.1 Review of BTV experimental infection studies

A review of data from BTV experimental infection studies was performed to yield a range of C_T_ values recorded over a 30-day BTV-infection period for bovines, ovines and caprines. Google scholar was used to source articles published since 2007 containing the following search parameters; “Bluetongue virus”, “infection”, “kinetics”, “experimental”, “pathological”, “real-time RT-PCR”. Articles were further screened for those reporting C_T_ values either in the article text or easily interpretable from figures. Eleven articles were chosen which represented a total of 12 experimental BTV infection studies in bovines (n = 4), ovines (n = 6) and caprines (n = 2). C_T_ values greater than 40 were ascribed as undetected (Undet.), therefore, the data compiled from the 12 experimental studies only considers C_T_ values lower than 40 (Table 1). The studies involved infection with BTV serotypes BTV-1 BTV-4, BTV-6, BTV-8 and BTV-26.

**Table 1:** Mean C_T_ values recorded during experimental BTV infection.

Representative	Bovine	Ovine	Caprine
dpi	Mean C_T_ value ± SD		
0	Undet. ± n.a.	Undet. ± n.a.	Undet. ± n.a.
1	34.66 ± 2.09	n.d. ± n.a.	n.d. ± n.a.
2	33.69 ± 1.57	34.55 ± 4.61	37.20 ± n.a.
3	34.42 ± 4.11	33.48 ± 3.13	n.d. ± n.a.
4	33.44 ± 4.07	26.86 ± 6.33	34.60 ± 1.53
5	28.97 ± 4.45	25.95 ± 4.10	n.d. ± n.a.
6	28.37 ± 3.55	23.35 ± 3.96	n.d. ± n.a.
7	25.36 ± 2.83	23.66 ± 3.13	23.90 ± 1.08
8	25.82 ± 3.87	23.92 ± 2.03	n.d. ± n.a.
9	26.49 ± 3.10	26.39 ± 1.94	22.30 ± 0.58
10	26.33 ± 3.66	25.69 ± 2.90	n.d. ± n.a.
11	28.00 ± n.a.	22.33 ± 2.69	21.27 ± 1.22
12	27.48 ± 3.63	26.46 ± 2.44	n.d. ± n.a.
13	26.13 ± 4.02	27.25 ± 1.77	n.d. ± n.a.
14	26.85 ± 3.29	25.28 ± 3.31	23.64 ± 0.62
15	n.d. ± n.a.	32.5 ± 2.12	n.d. ± n.a.
16	25.43 ± 1.80	26.94 ± 2.46	26.19 ± 2.89
17	28.26 ± 6.09	n.d. ± n.a.	n.d. ± n.a.
18	28.79 ± 3.96	27.83 ± 4.20	25.13 ± 2.18
19	n.d. ± n.a.	29.62 ± 3.58	n.d. ± n.a.
20	n.d. ± n.a.	26.75 ± 2.17	n.d. ± n.a.
21	28.91 ± 4.59	26.77 ± 3.15	27.54 ± 2.26
22	n.d. ± n.a.	29.72 ± 4.57	n.d. ± n.a.
23	27.83 ± 3.38	29.85 ± 2.53	28.71 ± 1.77
24	25.00 ± n.d.*	30.90 ± 3.59	n.d. ± n.a.
25	32.48 ± 5.19	30.51 ± 2.15	30.53 ± 0.32
26	n.d. ± n.a.	25.34 ± 1.66	n.d. ± n.a.
27	28.98 ± 0.59	30.90 ± 2.22	n.d. ± n.a.
28	n.d. ± n.a.	n.d. ± n.a.	29.23 ± 2.45
29	n.d. ± n.a.	25.17 ± 2.46	n.d. ± n.a.
30	27.46 ± 1.23	29.42 ± 1.30	30.00 ± 0.75

dpi. days post infection, Undet., undetected, n.d. no data available.

* based on a single C_T_ value, n.a. not applicable.

### 2.2 BTV isolates used for investigation and preparation of samples

Based on the current BTV situation in Europe, three recent European BTV serotypes (BTV-1, BTV-4 and BTV-8) were obtained from the Orbivirus Reference Collection held at The Pirbright Institute. Five-hundred microliters of the isolates: SPA2014/08 (BTV-1), ROM2014/06 (BTV-4) and FRA2015/01 (BTV-8) were diluted in BTV-negative bovine EDTA blood to yield a representative BTV C_T_ value for four stages of BTV infection: onset of infection, early viraemia, peak viraemia and late infection. To create the pools, 500 μl of the neat sample (the representative C_T_ value sample) was added to an appropriate volume of BTV-negative bovine EDTA blood to simulate pooling at different ratios of 1:2, 1:5, 1:10 and 1:20. A pooling ratio of 1:20 for the 0–2 dpi sample was not performed as it was assumed that the real-time RT-PCR assay would not detect BTV in these samples.

### 2.3 RNA extraction and real-time RT-PCR analysis

BTV RNA was extracted from 100 μl of EDTA blood using the KingFisher Flex automated extraction platform (ThermoFisher Scientific, Paisley, UK) and the MagVet Universal nucleic acid extraction kit (ThermoFisher) and RNA was eluted into 80 μl. Neat EDTA blood samples were extracted in triplicate while each of the pooled EDTA blood samples (1:2, 1:5, 1:10 and 1:20) were extracted and tested in ten replicates. Five microliters of BTV RNA was denatured at 95 °C for 5 min prior to analysis using two different real-time RT-PCR assays. The VetMAX assay was used according to the manufacturer’s instructions. The Hofmann et al., (2008) assay was performed using 15 μl of the reaction mix using the Express One-Step Superscript qRT-PCR kit (LifeTechnologies, Paisley, UK) containing 1 × reaction mix, 400 nM forward and reverse primers, 200 nM probe, 0.5 μl Rox, and 2 μl of enzyme mix in each well. Cycling conditions were as follows: reverse transcription at 50 °C for 15 min and 95 °C for 20 s min, and then 45 cycles of PCR, with each cycle consisting of 95 °C for 3 s, 56 °C for 30 s and 72 °C for 1 min. Real-time RT-PCR was performed on an Applied Biosystems 7500 Fast instrument (LifeTechnologies) using the fast ramp rates to provide results within 90 min for both assays.

### 2.4 Statistical analysis

Results of the pooling investigation were analysed using generalized linear models with binomial errors and a logit link function. The response variable was proportion of pools positive and explanatory variables were “assay” (Hofmann assay vs VetMAX assay), “C_T_ value of the positive sample”, “serotype” (BTV-1, BTV-4 or BTV-8) and “pooling ratio” (neat, 1:2, 1:5, 1:10, 1:20). The initial model was one including all explanatory variables as additive effects and two- and three-way interactions between “assay”, “C_T_ value” and “serotype”. Model selection proceeded by stepwise deletion of non-significant (P < 0.05) terms in the model, as judged by likelihood ratio tests. The final model was one in which all terms and interactions were significant (P > 0.05).

To extrapolate from the experimental results to provide guidelines for pooling, a simpler model was used in which the initial model included “assay”, “C_T_ value”, “serotype” and “dilution” as additive effects (i.e. no interactions were included). Model selection proceeded as described above.

## 3 Results

### 3.1 Review of C_T_ values recorded during BTV experimental infection studies

The mean C_T_ value for each dpi was calculated for three ruminant species: bovines, ovines and caprines based on the C_T_ values reported in BTV experimental infection studies (n = 12) (Table 1). In 6 studies, BTV was detected by 2 dpi and C_T_ values ranged from C_T_ 31.60 to C_T_ 38.61 with a mean C_T_ value at 2 dpi of 33.69, 34.55 and 37.20 for bovines, ovines and caprines, respectively. A mean C_T_ value for the period 0–2 dpi of 34.91 was calculated for all species. BTV was detected by 5 dpi in all remaining studies (n = 6) and C_T_ values ranged from C_T_ 20.83 to C_T_ 35.41. The mean C_T_ values recorded at 6 dpi were 28.37 and 23.35 for bovines and ovines, however, no data existed for caprines at this dpi. For the period 3–6 dpi, a mean C_T_ value of 29.73 was calculated for all species.

Peak viraemia was detected in all species between 7 to 12 dpi and C_T_ values ranged from C_T_ 18.30 to C_T_ 34.00. At peak viraemia, a mean C_T_ value of 26.58, 24.74 and 22.49 was obtained for bovines, ovines and caprines, respectively. A mean C_T_ value of 25.18 was calculated for all species between the period 7–12 dpi. BTV was detected post-peak viraemia (considered to be late infection; 13–30 dpi) for the duration of the experiments. The mean C_T_ value at 30 dpi for bovines, ovines and caprines was 27.46, 29.42 and 30.00, respectively. A mean C_T_ value of 28.14 was calculated for all species between the period 13–30 dpi. This information provided the rationale for creating 4 representative BTV-positive samples containing conservatively high C_T_ values; onset of infection: C_T_ 36 (0–2 dpi), early viraemia: C_T_ 33 (3–6 dpi), peak viraemia: C_T_ 27 (7–12 dpi) and late infection C_T_ 30 (13–30 dpi). The chosen C_T_ values were considered to represent a conservatively high C_T_ value and could also be easily generated in the laboratory.

### 3.2 Performance of real-time RT-PCR assays

C_T_ values achieved by both the Hofmann and VetMAX assays are shown in Table 2. The mean C_T_ values achieved by both real-time RT-PCR assays were within 1 C_T_ value of the intended representative C_T_ value. Using the Pearson correlation coefficient, C_T_ values achieved by both real-time RT-PCR assays were correlated for BTV-1 (r = 0.982; P < 0.001), BTV-4 (r = 0.987; P < 0.001) and BTV-8 (r = 0.881; P < 0.001). The mean C_T_ value difference between the Hofmann and VetMAX assays was 0.24, −0.29 and −0.27 for BTV-1, BTV-4 and BTV-8, respectively however, this was not found to be significant (P = 0.66). Both assays detected BTV in the peak viraemia sample at all pooling ratios and in all replicates. In the late infection sample, a minimum of a 94% detection rate was found for all pooling ratios using the VetMAX assay. The onset of infection sample (C_T_ 36; 0–2 dpi.) when tested neat, did not yield C_T_ values in all replicates irrespective of the real-time RT-PCR assay used. In general for C_T_ values >33, an increasing pooling ratio approached the limit of detection for both assays. Both assays demonstrated a greater sensitivity for BTV-8 than BTV-1 or BTV-4.

**Table 2:** C_T_ values determined for neat and pooled EDTA blood samples.

		Hofmann et al. (2008) assay; mean C_T_ value ± SD (number positive)					VetMAX assay; mean C_T_ value ± SD (number positive)				
BTV serotype	dpi	Neat	Pooled 1:2	Pooled 1:5	Pooled 1:10	Pooled 1:20	Neat	Pooled 1:2	Pooled 1:5	Pooled 1:10	Pooled 1:20
BTV-1	0 to 2	35.39 ± 0.59 (2/3)	36.06 ± 0.87 (4/10)	37.93 ± 0.53 (3/10)	Undet. ± n.a. (0/10)	n.d. ± n.a. (n.d.)	35.35 ± 0.45 (2/3)	37.94 ± 1.34 (5/10)	Undet. ± n.d. (0/10)	38.03 ± n.a. (1/10)	n.d. ± n.a. (n.d.)
	3 to 6	33.26 ± 0.38 (3/3)	34.57 ± 1.00 (10/10)	36.1 ± 1.30 (7/10)	38.04 ± 1.54 (5/10)	37.76 ± n.a. (1/10)	32.87 ± 0.33 (3/10)	34.76 ± 0.80 (9/10)	36.16 ± 1.34 (9/10)	37.69 ± 0.84 (7/10)	37.75 ± n.a. (1/10)
	7 to 12^a^	27.25 ± 0.01 (3/3)	28.47 ± 0.25 (10/10)	29.98 ± 0.22 (10/10)	31.3 ± 0.22 (10/10)	33.13 ± 0.82 (10/10)	26.93 ± 0.09 (3/3)	27.96 ± 0.10 (10/10)	29.47 ± 0.12 (10/10)	30.75 ± 0.18 (10/10)	32.63 ± 0.80 (10/10)
	13 to 30	30.33 ± 0.15 (3/3)	31.27 ± 0.21 (10/10)	33.19 ± 0.47 (10/10)	35.11 ± 1.51 (10/10)	37.46 ± 3.21 (10/10)	29.67 ± 0.37 (3/3)	31.03 ± 0.16 (10/10)	32.64 ± 0.39 (10/10)	35.09 ± 0.92 (10/10)	35.84 ± 1.04 (10/10)

BTV-4	0 to 2	37.65 ± 3.36 (3/3)	38.92 ± n.a. (1/10)	39.66 ± n.a. (1/10)	Undet. ± n.a. (0/10)	n.d. ± n.a. (n.d.)	37.83 ± 1.00 (3/3)	37.11 ± 0.80 (6/10)	37.51 ± 0.75 (4/10)	Undet. ± n.a. (0/10)	n.d. ± n.a. (n.d.)
	3 to 6	32.67 ± 0.84 (3/3)	35.19 ± 1.41 (10/10)	37.55 ± 1.33 (3/10)	36.15 ± 1.34 (3/10)	37.09 ± n.a. (1/10)	33.28 ± 0.23 (3/3)	34.45 ± 1.13 (10/10)	35.63 ± 0.95 (9/10)	36.05 ± 1.25 (9/10)	37.19 ± 0.66 (6/10)
	7 to 12^a^	25.53 ± 0.10 (3/3)	26.24 ± 0.20 (10/10)	27.92 ± 0.17 (10/10)	28.97 ± 0.32 (10/10)	30.52 ± 0.76 (10/10)	26.61 ± 0.70 (3/3)	27.56 ± 0.08 (10/10)	28.97 ± 0.24 (10/10)	30.21 ± 0.41 (10/10)	31.35 ± 0.51 (10/10)
	13 to 30	28.63 ± 0.35 (3/3)	29.84 ± 0.38 (10/10)	31.32 ± 0.41 (10/10)	33.4 ± 1.32 (10/10)	34.49 ± 0.96 (10/10)	29.6 ± 0.20 (3/3)	30.8 ± 0.40 (10/10)	32.06 ± 0.34 (10/10)	33.86 ± 0.80 (10/10)	34.37 ± 0.79 (10/10)

BTV-8	0 to 2	34.34 ± 0.29 (3/3)	35.59 ± 1.21 (4/10)	36.54 ± 1.05 (3/10)	36.63 ± 0.90 (2/10)	n.d. ± n.a. (n.d.)	37.05 ± 0.32 (2/3)	37.86 ± 0.92 (8/10)	37.52 ± 1.56 (6/10)	38.74 ± 0.01 (2/10)	n.d. ± n.a. (n.d.)
	3 to 6	34.38 ± 0.70 (3/3)	35.63 ± 0.76 (8/10)	36.81 ± 0.52 (2/10)	35.39 ± 0.71 (3/10)	37.55 ± n.a. (1/10)	32.87 ± 1.05 (3/3)	33.76 ± 0.80 (10'10)	35.52 ± 1.54 (10/10)	35.86 ± 1.39 (7/10)	37.01 ± 0.42 (3/10)
	7 to 12^a^	27.94 ± 0.24 (3/3)	29.13 ± 0.14 (10/10)	30.62 ± 0.54 (10/10)	31.1 ± 0.40 (10/10)	32.53 ± 0.52 (10/10)	28.92 ± 0.17 (3/3)	30.32 ± 0.21 (10/10)	32 ± 0.65 (10/10)	32.64 ± 0.46 (10/10)	34.18 ± 0.81 (10/10)
	13 to 30	31.38 ± 0.29 (3/3)	32.11 ± 0.41 (10/10)	33.8 ± 1.22 (10/10)	35.16 ± 1.15 (10/10)	36.23 ± 2.70 (08/10)	30.45 ± 0.27 (3/3)	31.25 ± 0.81 (10/10)	33.15 ± 1.02 (10/10)	34.86 ± 1.84 (9/10)	36.63 ± 1.81 (9/10)

dpi. Days post infection, n.d. no data available, n.a. not applicable.

a Peak viraemia.

### 3.3 Analysis of pooling data

The final model included “assay”, “C_T_ value”, “serotype” and “dilution” as fixed effects together with interactions between serotype and C_T_ value and interactions between serotype and assay. The probability of a pool testing positive was higher for the VetMAX assay compared with the Hofmann assay for BTV-4 and BTV-8 (BTV-4, odds ratio (OR): 10.0; BTV-8, OR: 4.0), but not for BTV-1. This indicated that the VetMAX assay is more sensitive than the Hofmann assay for BTV-4 and BTV-8, but not for BTV-1 (where the performance of both assays was comparable). The probability of a pool testing positive decreased with pooling ratio. In addition, pools were less likely to be positive as the C_T_ value of the initial sample increased, with the rate of decrease higher for BTV-1 and BTV-4 compared with BTV-8 (BTV-1, OR: 0.29; BTV-4, OR: 0.25; BTV-8, OR: 0.43).

### 3.4 Predicted impact of pooling EDTA blood samples to detect BTV

To provide more generic guidelines for pooling, a simpler model was fitted to the data which included “assay”, “C_T_ value” and “dilution” as significant (P < 0.001) fixed effects. Using this model there was no significant (P = 0.83) effect of BTV serotype. The predicted probabilities of a pool testing positive for different stages of BTV infection (C_T_ values) and pooling ratios are shown in Table 3 for the Hofmann and VetMAX assays.

**Table 3:** Estimated probability (%) of detection for different pooling ratios dependent on the C_T_ value of a single positive sample.

		Probability (%) of detection of a single positive animal			
Assay	Pooling ratio	C_T_ 27	C_T_ 30	C_T_ 33	C_T_ 36
		7–12 dpi	13–30 dpi	3–6 dpi	0–2 dpi
Hofmann	neat	100	99.94	98.69	76.14
	1:2	99.98	99.61	91.58	31.48
	1:5	99.92	98.13	68.86	8.54
	1:10	99.78	94.95	44.25	3.24
	1:20	99.08	82.02	16.16	0.81

VetMAX	neat	100	99.99	99.65	92.26
	1:2	100	99.90	97.60	63.19
	1:5	99.98	99.49	89.21	25.88
	1:10	99.94	98.60	74.79	11.13
	1:20	99.75	94.46	41.87	2.95

The model indicated that when the C_T_ value of the individual positive sample is low (C_T_ 27 or C_T_ 30) as occurs at peak viraemia and late infection (7–30 dpi), pooling of EDTA blood samples has little impact on the probability of the pool being detected positive. However, if the C_T_ value of the individual positive sample is higher (0–6 dpi: C_T_ 33 or C_T_ 36) as occurs at early stages of BTV infection, pooling of samples increases the probability of not-detecting a single infected animal in a pool. This is particularly the case for the onset of infection sample (C_T_ 36) where pooling at a 1:2 ratio reduced the probability of detecting a single infected animal.

## 4 Discussion

The aim of this study was to determine the most appropriate pooling ratio for EDTA blood samples to detect BTV using the most sensitive and commonly used real-time RT-PCR assays in the EU. Pooling of EDTA blood samples, subsequent extraction and real-time RT-PCR can increase the throughput of laboratories whilst also reducing reagent costs. This can be beneficial in an outbreak scenario where a large number of samples may need to be analysed from different locations. In our study, we created BTV-positive blood samples that were representative of blood samples collected at various time-points during experimental BTV infection as it was not possible to obtain material from experimental studies. We then applied a pooling ratio and following RNA extraction, analysed samples using two of the most commonly employed real-time RT-PCR assays in the EU as established during 2016 PT organised by the EURL for Bluetongue. A statistical model was then used to generate probabilities for detecting a single BTV-positive animal in a pool using these real-time RT-PCR assays.

Based on a review of BTV experimental infection studies, BTV infection kinetics for different ruminant species display a similar pattern: BTV can be detected by 5 dpi and for up to 30 dpi using real-time RT-PCR. We ascribed a representative C_T_ value to 4 stages of BTV infection: onset of infection (C_T_ 36, 0–2 dpi), early viraemia (C_T_ 33, 3–6 dpi), peak viraemia (C_T_ 27, 7–12 dpi) and late infection (C_T_ 30, 13–30 dpi) for bovine, caprine and ovine species. Due to the lack of material from experimental BTV infection studies, we created spiked EDTA blood samples containing BTV at C_T_ values deemed to represent a conservatively high C_T_ value. C_T_ values at the lower range would likely be detected by a well-optimised real time RT-PCR assay irrespective of the pooling ratios investigated in our study. Thus we considered an upper-range C_T_ value as the representative C_T_ value for the four stages of BTV infection; onset of infection, early viraemia, peak viraemia and late infection.

The literature reviewed for this study involved experimental infection with BTV-1, BTV-4, BTV-6, BTV-8, and BTV-26 and covered a variety of real-time RT-PCR assays. Our study involved three BTV serotypes, BTV-1, BTV-4 and BTV-8 which have been the most widely-circulating BTV serotypes in Europe since 2014, and thus were considered to provide an adequate representation of the BTV serotypes threatening wider distribution throughout Europe.

Using our representative peak viraemia (7–12 dpi) and late infection (13–30 dpi) samples, we found that pooling of up to 1:20 yields a high probability of detecting a single positive animal within a pool, especially for the VetMAX assay. For each BTV serotype investigated, the peak viraemia sample when pooled at different ratios, yielded C_T_ values in all ten replicates for both assays. Therefore, pooling at ratios of 1:20 would be acceptable for surveillance (and also to determine herd prevalence) in countries where BTV is endemic. The late infection sample when pooled at all ratios yielded a high probability of detecting BTV in the pool. An early study using conventional RT-PCR found that BTV is detectable up to 20 weeks post infection (Maclachlan et al., 1994). As real-time RT-PCR technologies offer increased sensitivity, it is reasonable to conclude that animals in late infection would similarly be detected using the well-optimised real-time RT-PCR assays considered in our study. This supports the rationale that a pooling ratio of 1:10 or 1:20 would be particularly suitable in supporting declaration of freedom of disease (following a vaccination program) since early-infections would not be occurring at this time (Vandenbussche et al., 2008b).

The onset of infection and early viraemia samples indicated that pooling of EDTA blood samples would significantly reduce the probability of detection of a single BTV-positive animal within the pool and it is quite likely to lead to false negative results. We found that samples at earlier stages of infection, when pooled, approached the limit of detection of both assays. These results are in agreement with the earlier reports by Batten et al. (2009) and Vandenbussche et al. (2008a) whereby pooling of samples taken from animals at early stages of viraemia, is not recommended. However, animals at these early stages of BTV infection present a challenge to real-time RT-PCR assays as from our literature review, only half of the studies detected BTV at 0–2 dpi in unpooled EDTA blood samples. In the UK, animals imported from areas within the EU BT restriction zone must be sampled within 7–10 days post-import. Were infection of the ruminant to occur on the day of import in the originating country, the day of sampling within the UK would lie close to or within the peak of viraemia as indicated in the reviewed experimental studies, thus making pooling of samples a suitable option.

In October 2017, a consignment of 32 animals imported into the UK from France was found to contain 7 BTV-positive animals through routine post-import testing (using a 1:5 pooling ratio) by the EURL for bluetongue (ProMED-mail, 2017). Following the initial analysis, EDTA blood samples were tested individually to yield C_T_ values within the range 27.14–32.54. We applied the greater pooling ratios investigated in this study (1:10, 1:20) to these field samples to support the conclusions arising from our model (data not shown). When these individual samples were tested using a pooling ratio of 1:10, BTV was detected in all replicates. Thus, the results from our study would support the use of a pooling ratio of 1:10 to detect a single infected animal within a pool, provided that the animal was sampled in accordance with the guidelines specified by the competent authority. In the UK, EDTA blood samples for testing must be collected 7–10 days post-import: we found that this rationale allowed for the detection of BTV-positive animals imported from France in October 2017 and that the pooling ratio could be extended to 1:10.

The necessity for BTV surveillance programmes presents a challenge to EU member states competent authorities and to the testing laboratories, especially as multiple BTV serotypes (BTV-1, BTV-4, BTV-8, and BTV-16) are circulating within the EU (More et al., 2017). The individual testing of animals by real-time RT-PCR presents the most sensitive means to detect active BTV infection in animals. However, pooling of EDTA blood samples can be used to detect BTV in animals arising from BTV-endemic countries. In this instance, it would be most appropriate to apply such a pooling ratio (1:10) to animals arising from a single geographic location or herd, since the possibility of a single BTV-infected animal existing within a cohort would be unlikely. The results achieved by the assays investigated in our study would support the use of pooling as a cost mitigation strategy in three epidemiological scenarios.

• In countries where BTV is endemic, a pooling ratio of 1:20 would yield a high probability of detecting BTV since it is likely that a large proportion of animals would be at various stages of BTV viraemia.
• To declare freedom of disease (following an appropriate vaccination strategy), a pooling ratio of 1:10 or 1:20 would provide a high probability of detecting BTV since new infections should not be occurring at this time.
• For the post-import testing of animals originating from areas within the EU BTV restriction zone, a pooling ratio of 1:10 is sufficient to detect BTV when animals are sampled 7–10 days post import.
